# The list of species registered in taiga meadow community during succession under enhanced radioactive background

**DOI:** 10.1016/j.dib.2018.04.121

**Published:** 2018-05-05

**Authors:** B. Gruzdev, T. Maystrenko, E. Belykh, A. Rybak

**Affiliations:** Institute of Biology, Komi Scientific Center, Ural Division RAS, Kommunisticheskaya 28, 167982 Syktyvkar, Russia

**Keywords:** Restoration of vegetation cover, Meadow community development, Taiga zone, Radioactive contamination

## Abstract

The data presented in this article are related to the research article entitled “The succession of the plant community on a decontaminated radioactive meadow site” (T. Maystrenko, B. Gruzdev, E. Belykh, A. Rybak, 2018) [1]. Primary data on floristic studies of meadow community development in taiga zone on the site contaminated with naturally occurring radionuclides are shown. The information given allows to follow a process of appearance and exclusion of high plant species from the pioneer step of succession up to stable community formation and to compare the structure and composition of meadow communities formed on territories with the enhanced and natural radioactivity background.

**Specifications Table**

**Table:** 

Subject area	Environmental sciences
More specific subject area	Radioecology, floristic studies
Type of data	Table, figures, text file
How data was acquired	Floristic analyses
Data format	Raw and Analyzed
Experimental factors	Does not apply
Experimental features	Floristic analysis of meadow community in taiga zone was performed to follow succession steps from 1962 up to 2012 year on the radioactively contaminated site in. Comparison of the community developed *de novo* with the surrounding vegetation growing under normal radioactive background was made in 2012.
Data source location	Around Vodny settlement, Komi Republic, Russia (63°31′ N, 53°26′ Е)
Data accessibility	Maystrenko T., Gruzdev B., Belykh E., Rybak A.
	The succession of the plant community on a decontaminated radioactive meadow site

**Value of the data**

• Data presented allows to follow the meadow community formation in the north taiga subzone under technogeneous contamination.
• A process of appearance and exclusion of high plant species from the pioneer step of succession up to stable community formation is shown.
• The structure and the composition of meadow community in the North taiga zone on territories with the enhanced and natural radioactivity background could be compared with plant communities formed in other ecological conditions.

## 1 Data

**Fig. 1:**
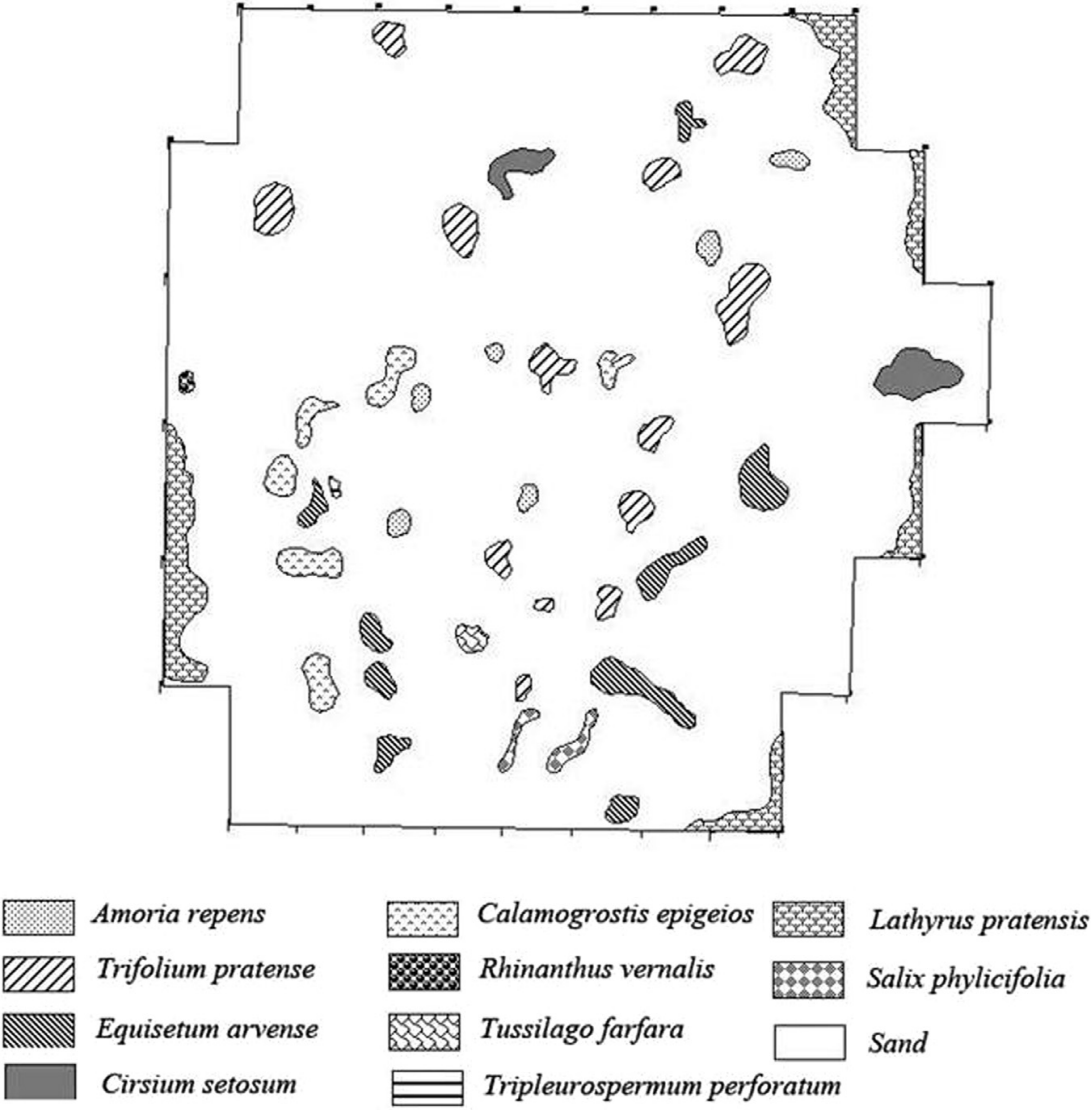
The pattern of dominating vascular plant species on the studied site in 1965 year, three years after decontamination.

**Fig. 2:**
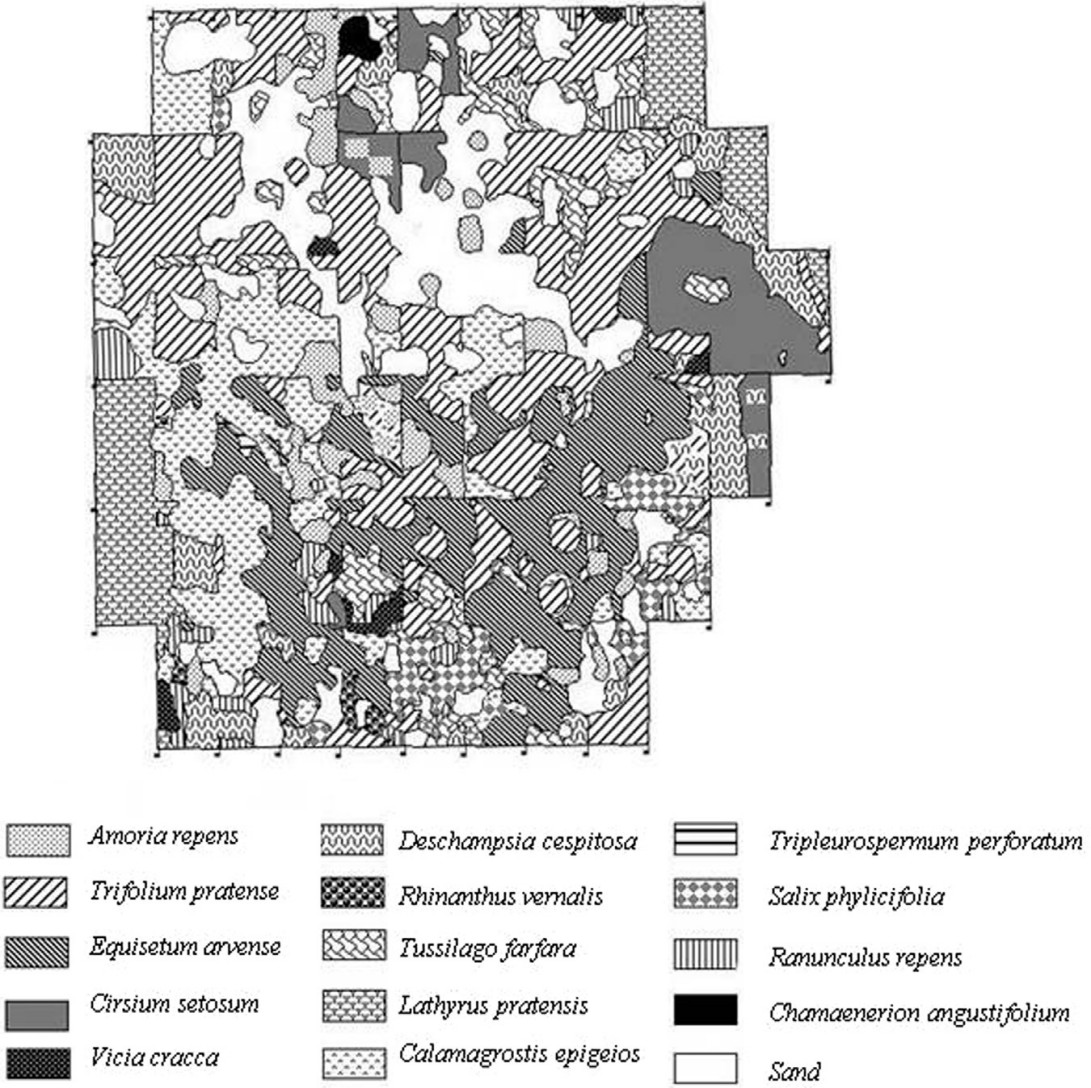
The pattern of dominating vascular plant species on the studied site in 1967 year, five years after decontamination.

**Fig. 3:**
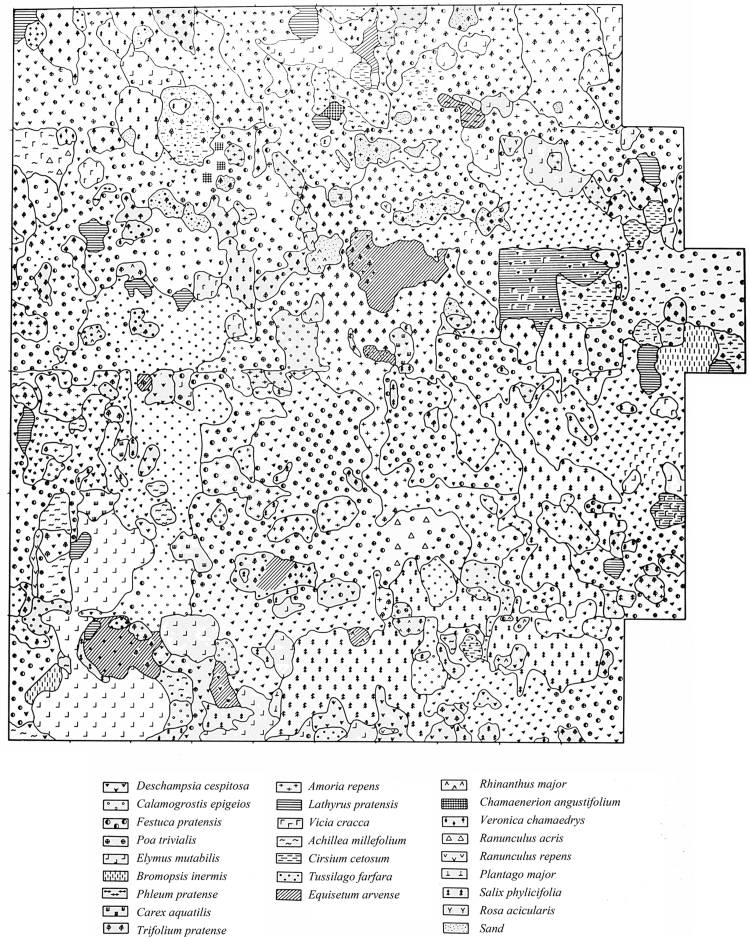
The pattern of dominating vascular plant species on the studied site in 1970, eight years after decontamination.

Data on high plant species presence/absence during long time observation of overgrowing of the radioactively contaminated site are given in Table 1 and Fig. 1, Fig. 2, Fig. 3. The site contamination was resulted from the ^226^Ra production plant activity; detailed description of radiation situation during the whole observation period presented in [1]. The first floristic analysis of the site was performed in 1962 year, four years after the termination of the commercial extraction of ^226^Ra. Then in 1962 the area was deactivated with filling with sand and gravel mixture. Short-term decrease in dose rate of γ-irradiation in the air and activity concentration of radionuclides in the root-inhabited soil layer up to background level had happened, but gradual increase in those values observed with vegetation cover development.

**Table 1:** The list of species registered on the contaminated site studied during succession and on the reference site at present.

No	Species	Family	Species presented									Longitudinal group	Latitudinal group	Ecological group	Life form	Life span
			on the contaminated site in the year of examination								On the reference site					
			1962^a^	1965	1967	1970	1976	1992^b^	2007	2012	2012					
**1**	**2**	**3**	**4**	**5**	**6**	**7**	**8**	**9**	**10**	**11**	**12**	**13**	**14**	**15**	**16**	**17**
1	Equisetum arvense L.	Equisetaceae	+	+	+	+	+	+	+	+		pz	hol	mp	h	p
2	Equisetum pratense Ehrh.	Equisetaceae									+	b	hol	mp	h	p
3	Equisetum sylvaticum L.	Equisetaceae							+			b	hol	mp	h	p
4	Pinus sylvestris L.	Pinaceae				+	+	+	+	+		b	ea	mhp	a	p
5	Agrostis gigantea Roth	Poaceae	+	+	+	+	+				+	b	ea	hmp	h	p
6	Agrostis stolonifera L.	Poaceae									+	b	ea	hp	h	p
7	Agrostis tenuis Sibth.	Poaceae						+		+		b	ea	mp	h	p
8	Alopecurus aequalis Sobol.	Poaceae			+							b	hol	hmp	h	a-b
9	Alopecurus pratensis L.	Poaceae	+	+	+	+	+	+	+	+	+	b	ea	hmp	h	p
10	Bromopsis inermis (Leyss.) Holub	Poaceae		+	+	+	+	+	+	+	+	b	e	hmp	h	p
11	Calamagrostis epigeios (L.) Roth	Poaceae	+	+	+	+	+		+	+	+	b	ea	xmp	h	p
12	Dactylis glomerata L.	Poaceae							+	+	+	b	ea	mp	h	p
13	Deschampsia cespitosa (L.) Beauv.	Poaceae	+	+	+	+	+	+	+	+	+	b	hol	hmp	h	p
14	Elymus fibrosus (Schrenk) Tzvel.	Poaceae			+	+	+					b	a	mp	h	p
15	Elymus mutabilis (Drob.) Tzvel.	Poaceae		+	+	+	+					b	s	mp	h	p
16	Elytrigia repens Nevski	Poaceae									+	b	ea	mp	h	p
17	Festuca ovina L.	Poaceae								+	+	pz	ea	mxp	h	p
18	Festuca pratensis Huds.	Poaceae		+	+	+	+	+	+	+	+	b	ea	mp	h	p
19	Festuca rubra L.	Poaceae		+						+	+	b	hol	mp	h	p
20	Hierochloë odorata (L.) Beauv.	Poaceae			+	+	+			+		b	hol	hmp	h	p
21	Milium effusum L.	Poaceae							+	+		b-n	hol	mp	h	p
22	Phalaroides arundinacea (L.) Rausch.	Poaceae	+		+					+	+	pz	hol	hp	h	p
23	Phleum pratense L.	Poaceae	+	+	+	+	+			+	+	b	ea	mp	h	p
24	Poa alpina L.	Poaceae				+	+					а-а	hol	mp	h	p
25	Poa annua L.	Poaceae	+									pz	c	mp	h	a
26	Poa nemoralis L.	Poaceae				+	+					b-n	e	mp	h	p
27	Poa palustris L.	Poaceae				+	+				+	b	hol	hp, hmp	h	p
28	Poa pratensis L.	Poaceae	+			+	+	+	+	+	+	b	hol	mp	h	p
29	Poa trivialis L.	Poaceae			+	+	+					b	e	mp	h	p
30	Carex acuta L.	Cyperaceae									+	b	ea	hp	h	p
31	Carex aquatilis Wahlenb.	Cyperaceae			+	+	+	+	+	+		b	hol	hp	h	p
32	Juncus compressus Jacq.	Juncaceae	+						+		+	b	ea	hp	h	p
33	Luzula multiflora (Ehrh.) Lej.	Juncaceae				+	+					b	ea	mp	h	p
34	Paris quadrifolia L.	Trilliaceae							+	+		b-n	e	mp	h	p
35	Populus tremula L.	Salicaceae			+	+	+				+	b	ea	mp	a	p
36	Salix carpea L.	Salicaceae	+					+	+	+		b	ea	mp	a	p
37	Salix dasyclados Wimm.	Salicaceae				+	+	+	+	+		b	ea	hp	a	p
38	Salix phylicifolia L.	Salicaceae		+	+	+	+	+	+	+	+	hyp	e	hp, hmp	s	p
39	Alnus incana (L.) Moench	Betulaceae			+	+	+	+	+	+		b	e	mp	a	p
40	Betula pubescens Ehrh.	Betulaceae			+	+	+	+	+	+		b	ea	mp, hmp	a	p
41	Urtica dioica L.	Urticaceae	+					+	+	+	+	pz	hol	mp	h	p
42	Bistorta major S.F. Gray	Polygonaceae				+	+					b	hol	hmp	h	p
43	Fallopia convolvulus (L.) A.Löeve	Polygonaceae	+									pz	hol	mp	h	a-b
44	Polygonum aviculare L.	Polygonaceae	+									pz	c	mp	h	a
45	Rumex crispus L.	Polygonaceae	+	+	+	+	+					b	hol	mp	h	p
46	Rumex confertus Willd.	Polygonaceae								+	+	pz	ea	mp	h	p
47	Chenopodium album L.	Chenopodiaceae	+	+		+	+	+				pz	c	mp	h	a
48	Cerastium holosteoides Fries	Caryophyllaceae	+	+	+	+	+		+			pz	hol	mp	h	p
49	Oberna behen (L.) Jkonn.	Caryophyllaceae			+	+	+			+		b	hol	mp	h	p
50	Silene tatarica (L.) Pers.	Caryophyllaceae									+	f-s	e	kmp	h	p
51	Stellaria holostea L.	Caryophyllaceae									+	b-n	e	mp	h	p
52	Stellaria graminea L.	Caryophyllaceae				+			+	+	+	b	ea	mp	h	p
53	Stellaria longifolia Muehl. ex Willd.	Caryophyllaceae									+	b	hol	mp	h	p
54	Stellaria media (L.) Vill.	Caryophyllaceae	+			+	+					pz	hol	mp	h	a-b
55	Aconitum septentrionale Koelle	Ranunculaceae				+	+		+	+		b	ea	mp	h	p
56	Ranunculus acris L.	Ranunculaceae	+		+	+	+	+	+	+	+	b	ea	mp	h	p
57	Ranunculus repens L.	Ranunculaceae	+		+	+	+		+	+	+	b	ea	mp, hmp	h	p
58	Thalictrum minus L.	Ranunculaceae			+	+	+	+	+	+		b	ea	mp	h	p
59	Thalictrum simplex L.	Ranunculaceae								+	+	b	ea	mp	h	p
60	Trollius europaeus L.	Ranunculaceae				+	+		+	+	+	b	e	mp	h	p
61	Paeonia anomala L.	Paeoniaceae	+							+	+	b	a	mp	h	p
62	Capsella bursa-pastoris (L.) Medik.	Brassicaceae	+									pz	hol	mp	h	a-b
63	Erysimum cheiranthoides L.	Brassicaceae	+	+	+	+	+					pz	hol	mp	h	a-b
64	Lepidium ruderale L.	Brassicaceae	+	+	+							pz	e	xmp	h	a
65	Thlaspi arvense L.	Brassicaceae	+									b	hol	mp	h	a-b
66	Barbarea vulgaris R. Br.	Brassicaceae							+		+	b	ea	mp	h	a
67	Ribes nigrum L.	Grossulariaceae	+			+	+					b	ea	hmp	s	p
68	Ribes hispidulum (Jancz.) Pojark.	Grossulariaceae								+		b	s	hmp	s	p
69	Alchemilla murbeckiana Bus.	Rosaceae				+			+	+	+	а-а	ea	hmp	h	p
70	Geum rivale L.	Rosaceae	+			+	+	+	+	+	+	b	ea	hp	h	p
71	Filipendula ulmaria (L.) Maxim.	Rosaceae	+		+	+	+	+	+	+	+	b	ea	hp	h	p
72	Fragaria vesca L.	Rosaceae	+	+	+						+	b	ea	mp	h	p
73	Potentilla intermedia L.	Rosaceae	+									b	e	xmp	h	p
74	Rosa acicularis Lindl.	Rosaceae		+	+	+	+	+	+	+	+	b	hol	mp	s	p
75	Rubus idaeus L.	Rosaceae	+				+	+	+	+	+	b	ea	mp	ss	p
76	Spiraea media F. Schmidt	Rosaceae	+						+	+		b	ea	mp	s	p
77	Lathyrus pratensis L.	Fabaceae	+		+	+	+	+	+	+	+	b	ea	mp	h	p
78	Trifolium pratense L.	Fabaceae	+	+	+	+	+	+	+	+	+	b	ea	mp	h	p
79	Amoria repens (L.) C. Presl	Fabaceae	+	+	+	+	+		+	+	+	b	ea	mp	h	p
80	Vicia cracca L.	Fabaceae	+		+	+	+		+	+	+	b	hol	mp	h	p
81	Vicia sepium L.	Fabaceae				+	+	+	+	+	+	b	ea	mp	h	p
82	Geranium sylvaticum L.	Geraniaceae				+	+	+	+	+	+	b	ea	mp	h	p
83	Geranium pratense L.	Geraniaceae									+	b	ea	mp	h	p
84	Viola tricolor L.	Violaceae	+			+	+		+		+	b	e	mp	h	a
85	Chamaenerion angustifolium (L.) Scop.	Onagraceae	+	+	+	+	+	+	+	+	+	b	hol	mp	h	p
86	Epilobium palustre L.	Onagraceae	+		+	+	+					b	hol	hmp	h	p
87	Hypericum maculatum Crantz	Hypericaceae									+	b	ea	mp	h	p
88	Anthriscus sylvestris (L.) Hoffm.	Apiaceae	+	+	+	+	+	+	+	+		b	ea	mp	h	p
89	Angelica sylvestris L.	Apiaceae								+		b	ea	mp	h	p
90	Conioselinum tataricum Hoffm.	Apiaceae									+	b	ea	hmp	h	p
91	Carum carvi L.	Apiaceae				+	+					b	ea	mp	h	p
92	Heracleum sibiricum L.	Apiaceae				+	+		+	+	+	b	ea	hmp	h	p
93	Pimpinella saxifraga L.	Apiaceae						+				b	ea	xmp	h	p
94	Vaccinium myrtillus L.	Ericaceae		+	+							b	hol	mp	us	p
95	Vaccinium vitis-idaea L.	Ericaceae		+	+							b	hol	mp	us	p
96	Galium boreale L.	Rubiaceae	+		+	+	+		+	+	+	b	ea	mp	h	p
97	Adoxa moschatellina L.	Adoxaceae	+						+			b	hol	hmp	h	p
98	Valeriana wolgensis Kazak.	Valerianaceae	+			+	+		+			b	e	hmp	h	p
99	Polemonium caeruleum L.	Polemoniacea									+	b	ea	mp	h	p
100	Galeopsis bifida Boenn.	Lamiaceae	+							+		b	ea	mp	h	a
101	Lamium album L.	Lamiaceae	+			+	+		+	+	+	b-n	hol	mp	h	p
102	Scutellaria galericulata L.	Lamiaceae									+	b	ea	mgp	h	p
103	Euphrasia frigida Pugsl.	Scrophulariaceae	+		+	+	+					b	e	hmp	h	a
104	Linaria vulgaris L.	Scrophulariaceae	+								+	b	ea	xmp	h	p
105	Melampyrum sylvaticum L.	Scrophulariaceae							+			b	e	mp	h	a
106	Melampyrum pratense L.	Scrophulariaceae								+	+	b	ea	mp	h	p
107	Rhinanthus vernalis (Zing.) Schischk. & Serg.	Scrophulariaceae	+	+	+	+	+		+	+		b	ea	mp	h	a
108	Veronica chamaedrys L.	Scrophulariaceae	+			+	+	+	+	+	+	b-n	hol	mp	h	p
109	Veronica longifolia L.	Scrophulariaceae	+		+	+	+		+	+	+	b	ea	hmp	h	p
110	Plantago lanceolata L.	Plantaginaceae									+	pz	ea	kmp	h	p
111	Plantago major L.	Plantaginaceae	+	+	+	+	+				+	pz	hol	mp	h	p
112	Plantago media L.	Plantaginaceae	+									b	ea	xmp	h	p
113	Achillea millefolium L.	Asteraceae	+	+	+	+	+	+	+	+	+	b	ea	mp	h	p
114	Antennaria dioica (L.) Gaertn.	Asteraceae		+	+	+	+					b	ea	xmp	h	p
115	Artemisia vulgaris L.	Asteraceae				+	+			+		b	hol	mp	h	p
116	Carduus crispus L.	Asteraceae	+	+	+	+	+		+			pz	ea	mp	h	p
117	Centaurea jacea L.	Asteraceae									+	f-s	e	kmp	h	p
118	Centaurea phrygia L.	Asteraceae									+	b	e	kmp	h	p
119	Cirsium heterophyllum (L.) Hill	Asteraceae						+	+	+		b	e	mhp	h	p
120	Cirsium palustre (L.) Scop.	Asteraceae									+	b	ea	hmp	h	p
121	Cirsium setosum (Willd.) Bess.	Asteraceae	+	+	+	+		+	+	+	+	pz	ea	mp	h	p
122	Crepis tectorum L.	Asteraceae		+		+	+					b	ea	mp	h	a-b
123	Crepis sibirica L.	Asteraceae								+	+	b	ea	mp	h	p
124	Erigeron acris L.	Asteraceae	+		+	+	+					b	hol	xmp	h	a
125	Hieracium caespitosum Dumort.	Asteraceae	+			+						b	ea	xmp	h	p
126	Hieracium umbellatum L.	Asteraceae									+	b	ea	mp	h	p
127	Leontodon autumnalis L.	Asteraceae	+									b	e	mp	h	p
128	Lepidotheca suaveolens (Pursh) Nutt.	Asteraceae	+	+		+						pz	hol	mp	h	a
129	Leucanthemum vulgare Lam.	Asteraceae									+	b	ea	mp	h	p
130	Ligularica sibirica (L.) Cass.	Asteraceae	+									b	ea	hp	h	p
131	Mulgedium sibiricum (L.) Cass. ex Less.	Asteraceae	+									b	hol	mp	h	p
132	Omalotheca sylvatica (L.) Sch. Bip. & F. Schultz	Asteraceae	+	+			+					b	hol	mp	h	p
133	Senecio vulgaris L.	Asteraceae	+									b	ea	mp	h	a
134	Sonchus arvensis L.	Asteraceae									+	pz	c	mp	h	p
135	Tanacetum vulgare L.	Asteraceae	+		+	+	+					b	ea	mp	h	p
136	Taraxacum officinale Wigg.	Asteraceae	+	+	+	+	+	+	+	+	+	b	ea	mp	h	p
137	Tripleurospermum perforatum (Merat) M. Lainz	Asteraceae	+	+	+	+	+					pz	e	mp	h	a-b
138	Tussilago farfara L.	Asteraceae	+	+	+	+	+		+	+		b	ea	mp	h	p

Latitudinal groups: b – boreal, hyp – hypoarctic, pz – polyzonal, b-n – boreal-nemoral, a-a – arcto-alpine, f-s – forest-steppe.

Longitudinal groups: hol – Holarctic, ea – Eurasian, e – European, s – Siberian, a – Asian, c – cosmopolitan.

Ecological groups: mp – mesophytes, hmp – hygromesophytes, xmp – xeromesophytes, mxp – mesoxerophytes, hp – hygrophytes, mhp – mesohygrophytes.

Life form: h – herbs, a – arboreal, s – shrub, ss – subshrub, us – undershrub.

Life span: p – perennial, a-b – annual-biennial, a – annual.

a examination of the site performed before decontamination.

b only species significant for the cenosis were included to the list this year.

Following observations of the community formation with plants from adjacent area were performed up to 2012 year. The list of species presented on the site at time of observation is given in Table 1. Floristic description of reference meadow community was made in 2012 to compare the community developed *de novo* with surrounding area characterized the background radiation level.

Figures show the pattern of dominating vascular plant species on the studied site in 1965 (Fig. 1), 1967 (Fig. 2) and 1970 (Fig. 3) years.

## 2 Experimental design, materials and methods

### 2.1 Floristic analysis

The development of plant community under dynamic radiation background was observed for 50 years. Registrations were performed in 1962, just before the deactivation and then in 1965, 1967, 1970, 1976, 1992, 2007 and 2012 years. Intervals among examinations depended on the succession steps. Also activity concentrations of radionuclides in soil and plants were traced during the observation period, detailed data presented in [1].

An auxiliary grid net containing 55 cells (5 × 10 m each) was made for the experiment on the contaminated site. Vascular plants were listed for each cell. Plant cover was mapped based on the data on dominating plant species in every square meter of the cell. A life form of each species from the list was assigned according to Raunkiær [2]. Geographical and ecological group was determined according to the regional classification [3]. Modern floristic analysis was based on comparing lists of species from geobotanical descriptions made on 100 m^2^ sampling plots at the former radium production plant and natural vegetation sites with background concentrations of radionuclides in the soil.

## References

[1] T. Maystrenko, B. Gruzdev, E. Belykh, A. Rybak, The succession of the plant community on a decontaminated radioactive meadow site, Journal of Environmental Radioactivity. 10.1016/j.jenvrad.2017.12.013. 29571956

[2] B.A. Bikov, Geobotaniks, Nauka KazSSR, Alma-Ata (in Russian), 1978.

[3] A.I. Tolmachev, (Ed.), Flora of the Northeast of European part of the USSR, Nauka, Leningrad. V. I–IV (in Russian), 1974.

